# Do coder characteristics influence validity of ICD-10 hospital discharge data?

**DOI:** 10.1186/1472-6963-10-99

**Published:** 2010-04-21

**Authors:** Deirdre A Hennessy, Hude Quan, Peter D Faris, Cynthia A Beck

**Affiliations:** 1 Department of Community Health Sciences, University of Calgary, 3rd Floor TRW Building, 3280 Hospital Drive NW, Calgary T2N 4Z6, Alberta, Canada; 2 Department of Psychiatry, University of Calgary, Foothills Medical Centre AW 259C, 2nd Floor Special Services Building, 1403- 29th Street NW, Calgary, Alberta T2N 2T9, Canada

## Abstract

**Background:**

Administrative data are widely used to study health systems and make important health policy decisions. Yet little is known about the influence of coder characteristics on administrative data validity in these studies. Our goal was to describe the relationship between several measures of validity in coded hospital discharge data and 1) coders' volume of coding (≥13,000 vs. <13,000 records), 2) coders' employment status (full- vs. part-time), and 3) hospital type.

**Methods:**

This descriptive study examined 6 indicators of face validity in ICD-10 coded discharge records from 4 hospitals in Calgary, Canada between April 2002 and March 2007. Specifically, mean number of coded diagnoses, procedures, complications, Z-codes, and codes ending in 8 or 9 were compared by coding volume and employment status, as well as hospital type. The mean number of diagnoses was also compared across coder characteristics for 6 major conditions of varying complexity. Next, kappa statistics were computed to assess agreement between discharge data and linked chart data reabstracted by nursing chart reviewers. Kappas were compared across coder characteristics.

**Results:**

422,618 discharge records were coded by 59 coders during the study period. The mean number of diagnoses per record decreased from 5.2 in 2002/2003 to 3.9 in 2006/2007, while the number of records coded annually increased from 69,613 to 102,842. Coders at the tertiary hospital coded the most diagnoses (5.0 compared with 3.9 and 3.8 at other sites). There was no variation by coder or site characteristics for any other face validity indicator. The mean number of diagnoses increased from 1.5 to 7.9 with increasing complexity of the major diagnosis, but did not vary with coder characteristics. Agreement (kappa) between coded data and chart review did not show any consistent pattern with respect to coder characteristics.

**Conclusions:**

This large study suggests that coder characteristics do not influence the validity of hospital discharge data. Other jurisdictions might benefit from implementing similar employment programs to ours, e.g.: a requirement for a 2-year college training program, a single management structure across sites, and rotation of coders between sites. Limitations include few coder characteristics available for study due to privacy concerns.

## Background

Administrative data including hospital discharge abstract data have been extensively used to examine health and health systems and have provided insights into health care practices. However, the validity of such administrative data for use in research has been questioned [1]. As a result substantial efforts are being made to thoroughly assess these data for quality [2-7]. Many investigators [3-5,8-10] have conducted validation studies of International Classification of Disease 9^th ^Revision Clinical Modification (ICD-9-CM) coding focusing on individual data items, certain clinical conditions and complications of substandard care. These studies have found that administrative data are accurately coded for severe or life-threatening conditions such as myocardial infarction and cancer, but that some non-specific conditions like rheumatologic disease, are less accurately coded. The introduction of the International Classification of Disease 10^th ^Revision (ICD-10) coding system, gave rise to additional validation studies which concluded that although the ranking in leading causes of death in the US [11] and trends in cause-specific mortality in Europe [12] changed between the two systems, coding validity by and large remained the same [13].

The quality of coded data is influenced by two major factors: first how clearly, precisely and completely health care workers (primarily physicians) document diagnoses and treatments in the patient chart and second, how accurately and consistently the charts are coded by health records coders. The mechanisms by which both of these processes affect the quality of administrative data remain unclear. However, the former (physician charting) is difficult to assess, being highly variable and dependent on many factors not elucidated in administrative data, such as communication between physician and patient and the experience and expertise of the physician [14]. The variability and validity of coding by health records technicians was the focus of this study. Specifically, variability and validity of coding of hospital discharge data in relation to coders' demographics and experience. Studies of coding validity have mostly attempted to assess the quality of coded medical information by relying on a code-recode or reabstraction methodology that determines the difference in coding outcomes between career coders and a panel of "expert" coders. These studies have invariably shown differences in coding between the career coders and the expert coders [15]. According to Iezzoni [1] and O'Malley [14], the process of assigning ICD codes is complicated, more of an art than a science. The many steps in the process of coding a diagnosis introduce numerous opportunities for error [14,16].

There may be many reasons, typically not elucidated in reabstraction studies, why interpretation of data can vary between coders. For instance Green and Benjamin [17], in their 1986 study of hospitals in Illinois, USA, found improved coding quality in hospitals that employed credentialed coders, provided feedback to enhance quality and offered continuing education to coding staff. More recently, Callen *et al*. [18] in comparing error rates of procedure coding between trained coders and operating theatre staff in an Australian hospital demonstrated high error rates among operating theatre staff, thereby highlighting the need for formal coding training. Additionally, Lorenzoni *et al*. [19], in a study of an Italian hospital, showed that a program of ongoing training, continuous monitoring and feedback proved to be successful in improving the quality of material abstracted from the medical chart. Furthermore, Santos *et al*. [20] elicited coding managers' perceptions and reported that coding quality could be improved by higher staffing levels, continuing education for coders and increased interaction with medical staff.

Previous studies [17-20] examining associations of coder characteristics and coding validity have generally assessed error rates only for individual data items and in addition did not specifically examine groups of similarly trained and credentialed coders. Our study was conducted to address the quality of coded administrative data by coders' characteristics related to employment and experience, and unfolded in two parts. First, we examined validity of coding in all coded health records across a large number of diagnoses and co-morbidities over 5 years to establish face validity of the coded data in terms of coder characteristics. Second, we investigated the validity of coding in relation to coder characteristics by assessing agreement between the coded discharge data and data obtained by chart review and reabstraction in a subset of our data. The goal was to provide evidence to inform quality improvement initiatives in coders' training.

## Methods

### Study Design

This was a descriptive study of ICD-10-Canadian Version (CA) coding validity in Calgary, Alberta, Canada, a city of approximately 1.2 million people. The study was approved by the Conjoint Health Research Ethics Board at the University of Calgary.

### Part 1: Assessment of face validity of coding

#### Data Sources and Linkage

**Figure 1:**
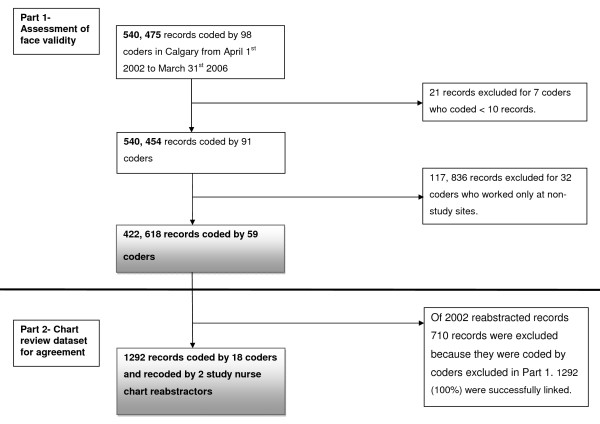
**Flow chart of record linkage**.

The health records coders described in this study are professionally trained and worked at four hospitals in Calgary. One coordinator supervises and manages the coding practice at the four sites to establish a consistent approach to coding. Each discharge record contained a unique identification number for each admission and coder and up to 16 coded diagnoses and 10 coded procedures. All the data in the discharge record was coded and no data was available in free text format. Using the coder identification number, hospital discharge records of patients admitted to Calgary hospitals between April 1^st ^2002 and March 31^st ^2007 were linked to coders' employment data (100% linkage), see Figure 1. Before linkage, coders working at Calgary facilities other than the four major hospitals were excluded (32 coders), as were coders that coded less than 10 records in total (7 coders). The final dataset for Part 1 of the study consisted of 422,618 discharge records coded by 59 coders. The coders excluded from this analysis were less often part-time employees (approximately 27% compared to 44%) and a higher proportion were high volume coders (27% compared to 20%).

#### Indicators of Face Validity

Face validity of coding was assessed by looking at 6 indicators in each record: (1) number of diagnoses, (2) number of procedures, (3) number of complications, (4) number of Z codes (which pertain to factors influencing health status and contact with health services) and (5 and 6) number of unspecified diagnoses (those codes ending in .8 or .9). Theoretically, a greater number of diagnoses, procedures, complications and Z codes noted in the discharge record provide a more detailed picture of the patient. Conversely, a greater number of unspecified codes in the record could indicate lack of detail and potentially lack of validity.

In addition, the mean number of diagnoses coded per admission was determined for different levels of complexity of the major condition. We expected that the number of co-morbid diagnoses would increase with the complexity of the major condition. For this analysis we identified 6 common conditions of varying complexity which were coded as the major diagnosis.

The coder characteristics that were assessed included volume of coding (high volume vs. low volume), employment status (full-time vs. part-time), and hospital site (tertiary hospital vs. others). We defined high volume coders as those who were at the 80^th ^percentile or higher of coding volume. This corresponded to ≥13, 000 records coded during the study period. Low volume coders made up the balance. We also described the number of records coded by year and because temporal trends in coding have been previously observed [19]. We also hypothesized that there may be seasonal variations in coding due to time pressures on coders at certain times of the year; therefore we described the number of records coded by season.

### Part 2: Assessment of agreement between coded record and chart review

#### Data Sources and Linkage

To assess agreement, a subset of data was assembled from the dataset in Part 1. Two nurses, who had previously worked as coding professionals, reviewed the charts and reabstracted data on 32 predefined conditions from a random sample of 2002 inpatient charts, as previously described [13]. These 32 conditions make up two commonly used risk adjustment taxonomies, the Charlson and the Elixhauser methods [21-24]. These reabstracted data were linked to the Part 1 dataset using the unique identification number for each admission. Of 2002 reabstracted charts 710 records were excluded because they were coded by coders that were excluded in Part 1 of the study. The final dataset for Part 2 of the study consisted of 1,292 discharge records coded by 18 coders, see Figure 1.

Next, the same 32 predefined conditions that were reabstracted by the nurse reviewers, were defined as being present or absent in the coded hospital discharge abstract record using ICD-10 coding algorithms [25].

### Statistical Analysis

For assessment of face validity (Part 1), descriptive statistics were employed to report the mean number of diagnoses, procedures, complications, unspecified diagnoses (ie. diagnoses ending in 8 or 9) and Z codes. The kappa statistic was used to assess agreement between the coded hospital discharge record and chart review data [26]. Kappa values were compared for high vs. low volume coders, full-time vs. part-time coders and by hospital site, i.e. tertiary care centre vs. all other sites.

## Results

### Part 1: Assessment of face validity

A total of 422,618 discharge records were coded by 59 coders during the study period. On average each coder coded 7,163 records during the study period, see Table 1. The number of records coded increased steadily over time, but did not vary substantially by season, indicating a constant workload throughout the year with no obvious reduction in coding in the summer months. Coders were worked predominantly at site B, the tertiary care site (37%) and mostly worked full-time (56%). Low volume coders coded up to 12,587 records while high volume coders coded 13,039 to 24,631 records.

**Table 1:** Number of records coded and characteristics of coders in a Canadian Health Region from fiscal year 2002 to 2006

Total number of records	422,618
**Number of records coded per fiscal year**	
2002	69,613
2003	72,783
2004	77,643
2005	99,737
2006	102,842

**Number of records coded per season**	
Jan-Mar	105,626
Apr-June	107,638
Jul-Sep	103,690
Oct-Dec	105,664

**Total number of coders**	59

**Number of coders by site**	
Site A	6
Site B (tertiary)	22
Site C	15
Site D	16

**Number of coders by volume of coding**	
Low volume (< 13,000 records)	47
High volume (≥13,000 records)	12

**Number of coders by employment status**	
Full time	33
Part time	26

The mean number of diagnoses per record coded decreased over time from 5.2 in 2002/03 to 3.9 in 2006/07, see Table 2. Conversely, the number of records coded per year increased over the same period as seen in Table 1. When the data were stratified by season, no variation in coding across the 6 indicators of face validity was seen. Coders at the tertiary care site recorded the highest mean number of diagnoses, 5.0. No substantial variation was demonstrated over the other 5 indicators.

**Table 2:** Mean number of coded diagnoses, procedures, complications, unspecified and Z codes by year, season, site and coder characteristics

Variable	Diagnoses	Procedures	Complications	Z codes	Codes ending in 8	Codes ending in 9
**Total sample**	**4.3**	**1.3**	**0.2**	**0.6**	**0.4**	**0.7**

**Year (fiscal)**						
2002	5.2	1.4	0.3	0.7	0.5	0.8
2003	4.7	1.4	0.2	0.6	0.4	0.8
2004	4.1	1.3	0.2	0.5	0.3	0.7
2005	3.8	1.2	0.2	0.5	0.3	0.7
2006	3.9	1.3	0.2	0.6	0.3	0.6

**Season**						
Jan-Mar	4.2	1.3	0.2	0.6	0.4	0.7
Apr-June	4.3	1.3	0.2	0.6	0.4	0.7
July-Sep	4.3	1.3	0.2	0.6	0.4	0.7
Oct-Dec	4.2	1.3	0.2	0.6	0.4	0.7

**Site**						
A	3.9	1.3	0.2	0.3	0.5	0.8
B (tertiary)	5.0	1.5	0.3	0.6	0.5	0.7
C	3.8	1.2	0.1	0.7	0.3	0.5
D	3.9	1.1	0.1	0.5	0.3	0.7

**Volume of coding**						
Low volume	4.3	1.3	0.2	0.5	0.4	0.6
High volume	4.2	1.2	0.2	0.6	0.3	0.7

**Employment status**						
Part-time	4.0	1.4	0.2	0.7	0.3	0.6
Full-time	4.4	1.2	0.2	0.5	0.4	0.8

In relation to complexity of the major diagnosis, the mean number of diagnoses increased from 1.5 for a singleton born in hospital (simplest condition examined), to a maximum of 7.9 for diabetes with complications (most complex condition examined), see Table 3. The number of diagnoses coded did not vary substantially by the coder's employment status or coding volume. Coders working at the tertiary hospital coded substantially more diagnoses for both diabetes with and without complications, than did coders working at other sites.

**Table 3:** Mean number of coded diagnoses by complexity of main diagnosis, and by coding volume and employment status of coders and hospital level

Condition	Number (%) of cases	Mean number of coded diagnoses by coding volume		Mean number of coded diagnoses by employment status		Hospital level	
		
		Low	High	Part time	Full time	Non tertiary	Tertiary
***Most complex*** Diabetes with complications	2007 (0.5%)	7.9	7.9	7.7	7.9	7.0	9.5
Alcohol abuse	2511 (0.6%)	5.3	5.4	5.6	5.3	5.1	5.9
Diabetes uncomplicated	1802 (0.4%)	5.0	4.9	4.4	5.2	4.4	6.8
Depression	5860 (1.4%)	4.4	4.3	4.7	4.2	4.2	4.7
Calculus of ureter	3616 (0.9%)	1.5	1.7	1.5	1.7	1.6	1.4
Singleton born in hospital ***Least Complex***	38,307 (9.1)	1.3	1.6	1.5	1.2	1.6	1.1

### Part 2: Assessment of agreement between coded record and chart review

**Figure 2:**
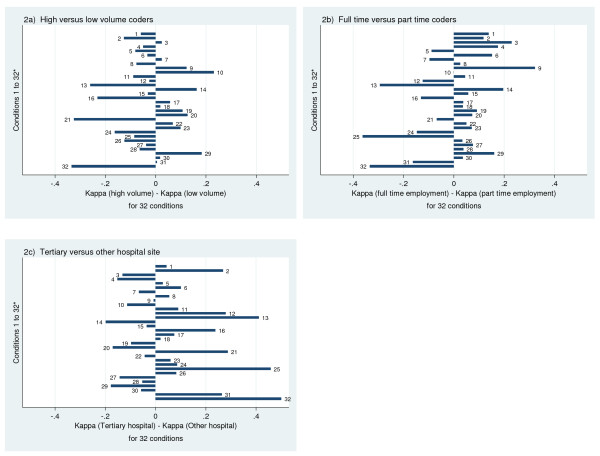
**a-c: Difference in agreement (Kappa) of coders' data with abstraction data by coder and hospital characteristics for Elixhauser **[22]**and Charlson **[21]**comorbidities, listed hereafter: 1) Myocardial infarction, 2) Cerebrovascular disease, 3) Rheumatic disease, 4) Dementia, 5) Cardiac arrhythmias, 6) Pulmonary circulation disorders, 7) Valvular disease, 8) Hypertension, 9) Hypothyroidism, 10) Lymphoma, 11) Solid tumour without metastasis, 12) Renal failure, 13) Blood loss anemia, 14) Deficiency anemia, 15) Coagulopathy, 16) Fluid and electrolyte disorders, 17) Weight loss, 18) Obesity, 19) Alcohol abuse, 20) Drug abuse, 21) Psychoses, 22) Depression, 23) Congestive heart failure, 24) Peripheral vascular disease, 25) Paralysis, 26) Chronic pulmonary disease, 27) Diabetes with complications, 28) Diabetes uncomplicated, 29) Peptic ulcer disease, 30) Metastatic cancer, 31) Liver disease, 32) HIV/AIDS**.

The kappa values ranged from a low of 0 for blood loss anemia in non-tertiary hospitals to a high of 1 for HIV in low volume coders and part time coders, and in tertiary hospitals, see additional file 1 (Additional Table S4). Of note, these extreme kappas occurred in low prevalence conditions. Since kappa is a function of prevalence, the prevalence of each condition as determined for both the hospital discharge data and the re-abstracted chart data is also presented in Additional file 1, Additional Table S4 [27]. For most conditions kappa was similar across employment status, coding volume and site. Differences in kappa that existed across coder characteristics did not show a consistent direction in favor of any particular coder characteristics, see Figure 2.

## Discussion

This study examined the relationship between the quality of coded administrative data health record coders' characteristics, specifically employment status and experience. First, we assessed the face validity of coded health records by coder characteristics across a large number of diagnoses over 5 years. Second, we investigated the agreement between the coded hospital discharge record and reabstracted chart review data in relation to coder characteristics in a subset of the data. Overall, our results suggest that these coder characteristics do not influence the validity of hospital discharge data. Notably, coding site (tertiary vs. other hospitals) is related to the number of diagnoses coded per record but not to the agreement (kappa) between coded record and chart review data.

This study assessed validity of coded data by looking at 6 indicators of face validity. We theorized that a greater number of diagnoses, procedures, complications and Z codes in the discharge record would provide a more detailed picture of the patient, while a greater number of unspecified codes in the chart would indicate lack of detail. Our results demonstrate that a greater number of diagnoses were coded by coders working at a tertiary care centre, while the number of diagnoses coded was remarkably similar across employment status (full-time vs. part-time) and volume (high volume vs. low volume).

Coding variation by site has also been described by Lorence *et al*., [28] who recorded lower oncology coding error rates in rural areas compared to metropolitan areas in the USA. Besides rural location other site related organizational factors are thought to influence coding validity. In a recent mixed methods study by Santos *et al*., [20] structural features related to the physical setting of the workplace and the coders' proximity to coding managers and clinicians were identified as influencing coding quality in the qualitative analysis. However, the quantitative portion of the Santos study did not reveal any significant relationship between physical location of coders and quality of coding. Interestingly Rangachari [29] showed that large teaching hospitals performed poorly compared to small, rural, non-teaching hospitals when it came to coding accuracy. While we did not specifically record coding errors in this study, a greater number of diagnoses were coded at the tertiary care centre than at other sites in our region. This could be explained by variation in structural features of the Health Records Department at the 4 sites as described above or in other organizational factors that we did not interrogate in this study, such as level of staffing and availability of resources. Another potential explanation for our result may be differences in the severity of illness of patients admitted to the various hospitals in our region. The tertiary centre cares for more acutely ill patients and is a referral centre for trauma patients; therefore on average we would expect patient records to contain more diagnoses.

Santos *et al*. [20] have demonstrated that workforce issues may affect coding quality at times when coders may have to perform multiple tasks and have tight deadlines for their work. Our results showed that the coding workload is unevenly distributed among coders in Calgary, with approximately half the coders (29 coders) coding less than 10% of the records, while the other half (30 coders) coded the remaining 90% of the records (data not shown). Local coding managers have reported staff shortages at times during the study period [30]. However, our results do not indicate variation in coding validity by season, which would be expected if the Health Records Department was consistently understaffed at specific times of the year or if coders had especially high workloads before the end of the fiscal year in March. Therefore our results do not support the premise that staff shortages are responsible for the uneven distribution of workload.

Besides variation in the mean number of diagnoses coded by hospital site, no other important variation in coding across the other 5 indicators of face validity was demonstrated. Moreover, our analysis by complexity of disease showed (as expected) that the number of diagnoses coded increased with increasing complexity of the major condition. Combined, these findings indicate coding consistency among coders, and provide the administrative data with face validity. In our region coding consistency is achieved through employing coders who are similarly trained and through a regionalized management structure [30]. Most coders undergo 2 years of training in an accredited college program that incorporates work experience. In addition, coders in the 4 major hospitals are managed under one Health Records Department and rotate between hospitals, thereby achieving consistent coding.

Our results demonstrate that while the volume of coding has risen over time, the number of diagnoses coded has decreased. Temporal trends in number of diagnoses coded have also been demonstrated by Lorenzoni *et al*. [19] in an Italian hospital. These authors showed an increase in diagnoses coded from 1994 to 1997 and attributed this improved trend to training, continuous monitoring and feedback to coders. While the trend to decreasing numbers of diagnoses coded over time in this study could point to less detailed coding and may seem to be of concern, especially to health researchers who look to administrative data to provide more rather than less detail, there are several other possible explanations for this result. First, the Health Records Department in our region may have been understaffed during the study period. This would be consistent with our result that the number of records increased as time went by. As increasing numbers of records are coded each year, coders may have less time to code each one and might therefore decrease the number of codes per record. However, as noted above there was no seasonal variation in mean number of diagnoses even at fiscal year end, which would argue against this explanation. Another possibility is that coding practices may have changed over time in relation to coding of diagnosis types. According to coding managers working at our institution, the coding of certain diagnoses designated as "type 3 diagnoses" is likely to have decreased over time [30]. These diagnoses do not satisfy the requirements to be considered a co-morbidity for administrative data purposes, and may or may not require treatment [31]. However, a change in coding of type 3 diagnoses is unlikely to affect assignment of records to a Case Mix Group (CMG) and as a result will impact minimally on the conclusions drawn by researchers from analyses of administrative data. Second, the documentation provided by physicians may have become less detailed overtime. This is difficult to assess but would not be related to coder characteristics. Third, the severity of illness of patients admitted to hospital in our region may have changed over time, again this would not be related to coder characteristics.

Finally, in investigating the validity of coding using reabstraction methodology in a subset of our data, we saw that for most conditions variations in kappa were similar across employment status, coding volume and site. Psychosis, HIV, Paralysis and Blood loss anemia were the conditions most often associated with wide variations in kappa across coder characteristics; however these differences in kappa did not show a consistent direction in favor of either high or low volume coders, full-time or part-time coders or hospital site.

This study has limitations. First, we had less information about health records coders' characteristics than we would have liked because of privacy concerns. Ideally we would have liked more comprehensive information, such as life time work experience and continuing education, because Santos and others have identified an important impact of ongoing education on coding quality [7,17,18,20,32]. Second, we did not include rural and small hospitals in our study, although variation in error rates of coding have been previously demonstrated in these settings [28,29]. Third, it is well known that inadequate documentation provided by clinicians may lead to poor quality coding; unfortunately it was not within the scope of this study to examine clinician documentation, which could have produced some of the variations in the coded data that was seen in this study.

On the other hand, a strength of this study was the use of reabstracted chart review data, which is considered by many researchers to be the "gold standard" when assessing coding validity [15]. In addition, we investigated coding validity in a very large number of hospital discharge abstracts, across a comprehensive list of major diagnoses.

## Conclusion

In summary, the quality of administrative data has implications for health records managers, health policy makers and for the reimbursement of healthcare expenditures as well as for academic researchers. Our study provided a comprehensive analysis of coding validity by year, coder characteristics and disease complexity. Our results suggest that coder characteristics do not influence the validity of hospital discharge data and that coding can be remarkably consistent. In Calgary this was achieved by employing coders with professional training and through a consistent management structure that included rotation of coders between sites. Other jurisdictions might benefit by employing these measures to ensure coding validity, thereby improving the quality of administrative data.

## Abbreviations

ICD-10-CA: International classification of disease version 10, Canadian version; ICD-9: International classification of disease version 9; USA: Untied States of American; HIV: Human immune deficiency virus; CMG: Case mix groups.

## Competing interests

The authors declare that they have no competing interests.

## Authors' contributions

DH: Made a substantial contribution to the concept and design of this study, she analyzed and interpreted the data, she drafted the manuscript, revised it critically and gave final approval for it to be published. HQ: Made a substantial contribution to the concept and design of this study, he acquired the data, was involved in interpretation of the data, critically revised the manuscript and gave final approval for it to be published. PF: Made a substantial contribution to the concept and design of this study, he was involved in interpretation of the data, critically revised the manuscript and gave final approval for it to be published. CB: Made a substantial contribution to the concept and design of this study, she was involved in interpretation of the data, critically revised the manuscript and gave final approval for it to be published.

## Pre-publication history

The pre-publication history for this paper can be accessed here:

http://www.biomedcentral.com/1472-6963/10/99/prepub

## Supplementary Material

Additional file 1 **Additional table S4**. Agreement (kappa) between coded record and chart review data for Elixhauser[22] and Charlson[21] co-morbidities, by coding volume and employment status of coders and coding site Click here for file
