# Supplementary material for: Do coder characteristics influence validity of ICD-10 hospital discharge data?
Source: BMC Health Serv Res. 2010 Apr 21;10:99. doi: 10.1186/1472-6963-10-99 (PMC2868845; doi:10.1186/1472-6963-10-99)
Supplement: Additional file 1 — Additional table S4. Agreement (kappa) between coded record and chart review data for Elixhauser[22] and Charlson[21] co-morbidities, by coding volume and employment status of coders and coding site [file 1472-6963-10-99-S1.DOC]

**Table 4: Agreement (kappa) between coded record and chart review data for Elixhauser(22) and Charlson(21) co-morbidities, by coding volume and employment status of coders and coding site**

| **Condition** | **Prevalence of condition**  **N (%)** | | **Kappa for low vs. high**  **coding volume** | | **Kappa for part time vs. full time employment status** | | **Kappa by hospital level** | |
| --- | --- | --- | --- | --- | --- | --- | --- | --- |
|  | **In hospital discharge data** | **In chart review data** | **Low** | **High** | **Part time** | **Full time** | **Non tertiary** | **Tertiary** |
| Myocardial infarction^1^ | 130 (10.06) | 191 (14.78) | 0.76 | 0.71 | 0.66 | 0.79 | 0.69 | 0.74 |
| Cerebrovascular disease^1^ | 63 (4.88) | 110 (8.51) | 0.63 | 0.51 | 0.52 | 0.64 | 0.36 | 0.63 |
| Rheumatic disease^1^ | 23 (1.78) | 42 (3.25) | 0.66 | 0.68 | 0.54 | 0.77 | 0.76 | 0.63 |
| Dementia^1^ | 25 (1.93) | 34 (2.63) | 0.77 | 0.72 | 0.63 | 0.81 | 0.83 | 0.68 |
| Cardiac arrhythmias^2^ | 125 (9.67) | 354 (27.40) | 0.45 | 0.37 | 0.46 | 0.38 | 0.38 | 0.41 |
| Pulmonary circulation  disorders^2^ | 17 (1.32) | 35 (2.71) | 0.35 | 0.32 | 0.25 | 0.40 | 0.27 | 0.38 |
| Valvular disease^2^ | 48 (3.72) | 121 (9.37) | 0.49 | 0.52 | 0.55 | 0.46 | 0.55 | 0.48 |
| Hypertension^2^ | 268 (20.74) | 380 (29.41) | 0.71 | 0.63 | 0.63 | 0.66 | 0.63 | 0.69 |
| Hypothyroidism^2^ | 39 (3.02) | 89 (6.89) | 0.49 | 0.61 | 0.35 | 0.67 | 0.56 | 0.56 |
| Lymphoma^2^ | 17 (1.32) | 19 (1.47) | 0.53 | 0.76 | 0.66 | 0.66 | 0.75 | 0.64 |
| Solid tumour without metastasis^2^ | 93 (7.20) | 116 (8.89) | 0.46 | 0.37 | 0.39 | 0.44 | 0.34 | 0.43 |
| Renal failure^2^ | 48 (3.72) | 45 (3.48) | 0.71 | 0.69 | 0.76 | 0.64 | 0.46 | 0.74 |
| Blood loss anemia^2^ | 12 (0.93) | 32 (2.48) | 0.41 | 0.15 | 0.44 | 0.14 | 0 | 0.4 |
| Deficiency anemia^2^ | 19 (1.47) | 31 (2.40) | 0.31 | 0.47 | 0.28 | 0.47 | 0.53 | 0.33 |
| Coagulopathy^2^ | 28 (2.17) | 126 (9.75) | 0.29 | 0.26 | 0.24 | 0.30 | 0.29 | 0.26 |
| Fluid and electrolyte disorders^2^ | 73 (5.65) | 172 (13.31) | 0.57 | 0.34 | 0.27 | 0.51 | 0.53 | 0.39 |
| Weight loss^2^ | 11 (0.85) | 78 (6.04) | 0.16 | 0.21 | 0.17 | 0.20 | 0.14 | 0.21 |
| Obesity^2^ | 23 (1.78) | 127 (9.83) | 0.26 | 0.28 | 0.26 | 0.28 | 0.25 | 0.29 |
| Alcohol abuse^2^ | 62 (4.80) | 126 (9.85) | 0.52 | 0.63 | 0.53 | 0.62 | 0.64 | 0.54 |
| Drug abuse^2^ | 39 (3.02) | 77 (5.96) | 0.44 | 0.57 | 0.48 | 0.56 | 0.59 | 0.43 |
| Psychoses^2^ | 22 (1.70) | 40 (3.10) | 0.82 | 0.50 | 0.71 | 0.64 | 0.51 | 0.79 |
| Depression^2^ | 70 (5.42) | 156 (12.07) | 0.48 | 0.55 | 0.48 | 0.54 | 0.55 | 0.50 |
| Congestive heart failure^1,2^ | 79 (6.11) | 104 (8.05) | 0.69 | 0.79 | 0.71 | 0.78 | 0.70 | 0.76 |
| Peripheral vascular disease^1,2^ | 33 (2.55) | 59 (4.57) | 0.64 | 0.47 | 0.48 | 0.57 | 0.63 | 0.49 |
| Paralysis^1,2^ | 24 (1.86) | 23 (1.78) | 0.61 | 0.53 | 0.22 | 0.68 | 0.79 | 0.43 |
| Chronic pulmonary disease^1,2^ | 95 (7.35) | 195 (15.09) | 0.66 | 0.54 | 0.56 | 0.59 | 0.53 | 0.62 |
| Diabetes with complications^1,2^ | 33 (2.55) | 30 (2.32) | 0.58 | 0.54 | 0.51 | 0.58 | 0.66 | 0.52 |
| Diabetes uncomplicated^1,2^ | 130 (10.06) | 154 (11.92) | 0.85 | 0.79 | 0.79 | 0.84 | 0.85 | 0.79 |
| Peptic ulcer disease^1,2^ | 5 (0.39) | 35 (2.71) | 0.13 | 0.31 | 0.14 | 0.30 | 0.35 | 0.17 |
| Metastatic cancer^1,2^ | 59 (4.57) | 63 (4.88) | 0.80 | 0.82 | 0.79 | 0.83 | 0.86 | 0.79 |
| Liver disease^1,2^ | 29 (2.24) | 56 (4.33) | 0.55 | 0.55 | 0.36 | 0.62 | 0.64 | 0.48 |
| HIV/AIDS^1,2^ | 3 (0.23) | 5 (0.39) | 1 | 0.67 | 1 | 0.67 | 0.49 | 1 |

^1^ Diagnosis is included in Charlson Index, ^2^ Diagnosis is included in Elixhauser co-morbidity method
